# JEPEG: a summary statistics based tool for gene-level joint testing of functional variants

**DOI:** 10.1093/bioinformatics/btu816

**Published:** 2014-12-12

**Authors:** Donghyung Lee, Vernell S. Williamson, T. Bernard Bigdeli, Brien P. Riley, Ayman H. Fanous, Vladimir I. Vladimirov, Silviu-Alin Bacanu

**Affiliations:** ^1^Department of Psychiatry, Virginia Institute for Psychiatric and Behavioral Genetics, ^2^Center for Biomarker Research & Personalized Medicine, Virginia Commonwealth University, Richmond, VA 23298, USA and ^3^Lieber Institute for Brain Development, Johns Hopkins University, Baltimore, MD 21205, USA

## Abstract

**Motivation:** Gene expression is influenced by variants commonly known as expression quantitative trait loci (eQTL). On the basis of this fact, researchers proposed to use eQTL/functional information univariately for prioritizing single nucleotide polymorphisms (SNPs) signals from genome-wide association studies (GWAS). However, most genes are influenced by multiple eQTLs which, thus, jointly affect any downstream phenotype. Therefore, when compared with the univariate prioritization approach, a joint modeling of eQTL action on phenotypes has the potential to substantially increase signal detection power. Nonetheless, a joint eQTL analysis is impeded by (i) not measuring all eQTLs in a gene and/or (ii) lack of access to individual genotypes.

**Results:** We propose joint effect on phenotype of eQTL/functional SNPs associated with a gene (JEPEG), a novel software tool *which uses only GWAS summary statistics* to (i) impute the summary statistics at unmeasured eQTLs and (ii) test for the joint effect of all measured and imputed eQTLs in a gene. We illustrate the behavior/performance of the developed tool by analysing the GWAS meta-analysis summary statistics from the Psychiatric Genomics Consortium Stage 1 and the Genetic Consortium for Anorexia Nervosa.

**Conclusions**: Applied analyses results suggest that JEPEG complements commonly used univariate GWAS tools by: (i) increasing signal detection power via uncovering (a) novel genes or (b) known associated genes in smaller cohorts and (ii) assisting in fine-mapping of challenging regions, e.g. major histocompatibility complex for schizophrenia.

**Availability and implementation:** JEPEG, its associated database of eQTL SNPs and usage examples are publicly available at http://code.google.com/p/jepeg/.

**Contact:** dlee4@vcu.edu

**Supplementary information:** Supplementary data are available at *Bioinformatics* online.

## 1 Introduction

Univariate analysis of genome-wide association studies (GWAS) has emerged as the main tool for identifying trait/disease-associated genetic variants (Burton *et al*., 2007). However, against initial expectations, most variants reported by complex trait GWAS are common single nucleotide polymorphisms (SNPs) with weak or moderate effect sizes, which account for only a small fraction of the overall phenotypic variation (Manolio *et al*., 2009). Presumably, most common causal variants are not detected in GWAS due to their small effect sizes (Yang *et al*., 2010). Therefore, to identify a greater number of causal variants, GWAS of (significantly) larger sample sizes is needed. However, such dramatic increase in sample size might be both time consuming and cost prohibitive.

One reasonable approach to increase the power to detect true association signals with small effect sizes is to use prior biological knowledge. For instance, researchers can prioritize the genetic variants by utilizing evidence/information of their impact on biological processes giving rise to the desired phenotypes. One such biological process is the regulation of gene expression, which is believed to have influenced human evolution and play an important role in diseases (Emilsson *et al*., 2008; Kudaravalli *et al*., 2009). Expression of most genes is influenced by expression quantitative trait loci (eQTLs), which were hypothesized to be prime candidates for causal variants affecting various phenotypes (Gaffney *et al*., 2012; Gilad *et al*., 2008). This hypothesis was subsequently empirically supported by the detection of significant eQTL enrichment among GWAS association signals (Nica *et al*., 2010; Nicolae *et al*., 2010). Recent studies making use of eQTL/functional information showed a boost in the detection rate of GWAS signals (Fehrmann *et al*., 2011; Nicolae *et al*., 2010; Schork *et al*., 2013). These functional approaches can take advantage of a diverse collection of databases/tools of functional annotations, which have become publicly available (ENCODE Project Consortium *et al*., 2012; Wang *et al*., 2010). For instance, the Encyclopedia of DNA Elements (ENCODE) Consortium has already catalogd huge amount of information on functional elements including gene expression, transcripts, transcription factor binding sites, chromatin, DNA methylation and histone modification patterns (ENCODE Project Consortium *et al*., 2012).

As mentioned earlier, to increase causal variant detection, signal enrichment in functional variants was used to justify prioritization/filtering procedures based on functional annotation (Schork *et al*., 2013). Recently, an improvement of this method was proposed. It first identifies functional annotations associated with phenotypes of interest and then uses them to prioritize SNPs (Pickrell, 2014). However, while useful, such an approach has the disadvantage of considering only the univariate effect of eQTL/functional SNPs. To leverage information from multiple SNPs, multi-SNP based association tests (Ehret *et al*., 2012; Wood *et al*., 2011; Yang *et al*., 2012) have been also proposed. Compared with univariate approaches, these methods certainly offer better detection power, but typically test all SNPs, not only the functional ones. Nonetheless, under the reasonable assumption that the causal pathways are mostly composed of functional SNPs, such approaches are likely to incur a power loss. Even more, under the same assumption, these approaches might diffuse [via linkage disequilibrium (LD)] the signals to relatively distant non-functional regions, which might add another layer of difficulty to any subsequent attempt to fine-map association signals.

Given the joint impact of eQTLs on gene expression, it is of great interest to multivariately analyse eQTL/functional SNPs in a gene. Nevertheless, for such a test, researchers need to impute a large fraction of these variants. Unfortunately, the commonly used genotype imputation methods need access to genetic data, which, unlike summary statistics, are not always available. Summary statistics-based imputation methods offer fast imputation with great accuracy (Lee *et al*., 2013; Pasaniuc *et al*., 2014; Pickrell, 2014). However, by default they impute all SNPs, not only the much less numerous eQTL SNPs. The unnecessary imputation of mostly unused SNPs makes these methods much more computer intensive than it is really necessary.

**Fig. 1.**
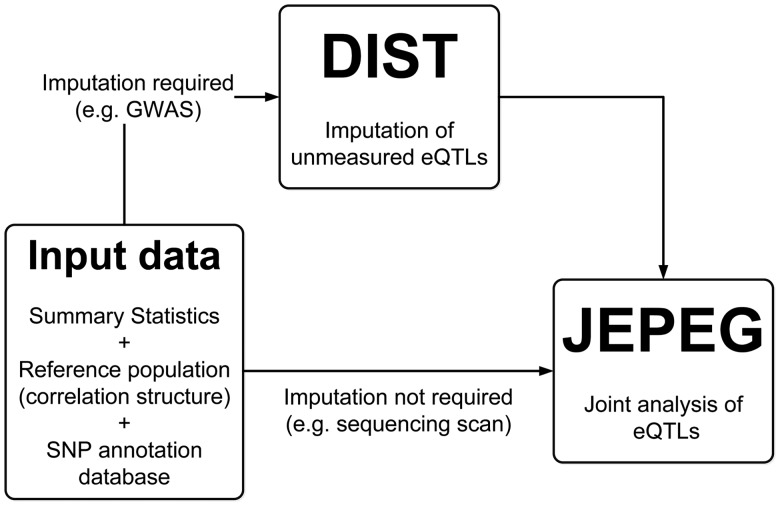
JEPEG flowchart. More detailed explanations on SNP annotation database, DIST, JEPEG and the reference population can be found in Sections 2.1–2.4

To improve over the state of the art, we propose joint effect on phenotype of eQTL/functional SNPs associated with a gene (JEPEG). JEPEG is an integrated method/software tool which uses only GWAS summary statistics to (i) rapidly and accurately impute summary statistics of unmeasured eQTL/functional SNPs and (ii) jointly test the effect of these (measured and imputed) functional SNPs associated with each gene in the genome. The associated software consists of four major components (Fig. 1): (i) an extensive database of eQTL/functional SNPs (Section 2.1), (ii) a module for directly imputing summary statistics of unmeasured eQTL/functional SNPs [i.e. Direct Imputation of summary STatistics (DIST) (Lee *et al*., 2013)] (Section 2.2), (iii) a module for testing the joint effect of all reliably measured/imputed functional SNPs associated with a gene (i.e. JEPEG) (Section 2.3) and (iv) reference population panels available/needed for both imputation and joint testing (Section 2.4).

## 2 Methods

### 2.1 SNP annotation database

To facilitate the pooling of SNPs effects within the same functional category (Section 2.3), the initial version of the database focuses on autosomal SNPs for which we can predict the direction and magnitude of allelic effects on gene expression. Because our applied analyses focus mainly on neuropsychiatric disease, the current version of the database is limited to SNPs affecting expression levels for brain-expressed genes. However, given that most genes are brain expressed, the use of this database for the analysis of non-psychiatric phenotypes might provide a tolerable first iteration in the absence of an eQTL database for the relevant tissue(s). The current SNP annotation database contains functional annotations for SNPs from the publicly available 1000 Genomes (1KG) (Altshuler *et al*., 2010) reference panel, Phase I version 3, which were subsequently screened *in* *silico* for an impact on the expression/function of brain-expressed genes. (The exception being the empirically derived cis- and trans-eQTL came from studies using smaller GWAS SNP panels.) The functional annotations include reference SNP cluster identifier (ID) (rsid), SNP location (chromosome and position), reference/alternative allele, associated gene ID, functional category, weight score, etc. Whenever available, we use human genome organization (HUGO) name for the gene having its expression/function affected by the eQTL/SNP entry. Conceptually, within each functional category, the weight score is a proxy measure for the predicted amount of *increase in* the expression of a gene brought on by the reference allele of its functional SNP. (Weight is negative when the reference allele is predicted to decrease gene expression.) Due to their diverse mode of acting on gene expressions, different functional categories might have different such proxy measures, e.g. free energy for the micro RNAs and deleteriousness score for protein function variants (Section 1 in Supplementary Data for more details). In the gene-based statistical analysis, the proxy measures act as weight scores that are used to combine, within each gene, the univariate summary statistics of measured and imputed SNPs within functional categories. Subsequently, these functional category statistics are combined in an overall gene level statistic.

In its current version, JEPEG uses SNPs belonging to six functional categories: (i) SNPs directly affecting protein function/structure encoded by a gene, i.e. protein function/structure (PFS) (e.g. stop codons), (ii) SNPs affecting expression of a gene by disrupting its transcription factor binding sites (TFBS), (iii) SNPs affecting the gene function by interrupting biogenesis of an miRNA (miRNA Structure), (iv) SNPs affecting miRNA–mRNA target interaction (miRNA Target) and non-categorized/empirically derived (v) cis- and (vi) trans-eQTLs. While PFS variants are not technically eQTLs, given the similarities between the two functional categories, we henceforth extend the definition of eQTLs to include PFS variants.

### 2.2 Direct imputation of summary statistics at unmeasured eQTLs

The SNP annotation database includes many functionally annotated SNPs that are not available in GWAS panels. Thus, before testing the multivariate effect of all functionally annotated SNPs affecting a gene, JEPEG imputes normally distributed statistics (two tailed Z-scores, henceforth called summary statistics) of the unmeasured functional SNPs. The imputation is achieved by employing DIST, one of our recently developed method/software which *directly* imputes summary statistics of unmeasured SNPs (Lee *et al*., 2013), i.e. without the need of a prior genotype imputation. When compared with the commonly used genotype imputation methods, e.g. IMPUTE2 (Howie *et al*., 2009), DIST was found to be of comparable accuracy and two orders of magnitude more efficient in terms of both running time and memory usage. Within JEPEG software, DIST module is silently run to impute summary statistics for unmeasured functional SNPs. Due to imputing only the less numerous set of unmeasured *functional* SNPs, the imputation module is much faster than the stand-alone DIST software. The high-quality imputation is achieved by applying the classical conditional expectation formula for multivariate normal variates using only (i) association summary statistics of reported markers within sliding windows with a fixed length and (ii) correlation matrix of homologous genotypes estimated from an external reference panel (e.g. 1KG).

In more detail, let *Z_u_* be the vector of *Z*-scores of unmeasured functional variants in the non-overlapping prediction window with a fixed length [0.1 mega base pairs (Mb) by default]. Denote as *Z_m_* the vector of Z-scores of all measured variants (including non-annotated measured variants) within the extended window (i.e. the prediction window with two fixed-length flanking regions (0.2 Mb by default)). Let *∑_u,m_* be the correlation matrix between the unmeasured and measured variants and *∑_m,m_* be the correlation matrix among the measured variants, which are both estimated from a reference panel. By using the classical conditional mean formula (Lee *et al*., 2013), *Z_u_* can be imputed as

$$Z_{u}=\sum_{u,m}{\left(\sum_{m,m}\right)}^{-\text{1}}Z_{m}.$$

The variance–covariance matrix (proxy imputation information measure) of *Z_u_* can be subsequently estimated as

$$I_{u}=\sum_{u,m}{\left(\sum_{m,m}\right)}^{-\text{1}}\sum_{u,m}{}^{\text{T}}.$$

To obtain imputation *Z*-scores with a variance of one, we normalize *Z_u_* using the square root of *I_u_* (Pasaniuc *et al*., 2014).

Due to the strongly correlated structure of the genotype data, the correlation matrix can be ill-conditioned and, therefore, result in a large standard error for the imputed *Z*-scores. The high variability of estimates can lead to poor accuracy and false positives. To circumvent the degradation of DIST performance, similar to Pasaniuc *et al.* (2014) and Pickrell (2014), we add a ridge adjustment (with a heuristical default value $\lambda =2/\sqrt{n}$, where *n* is the sample size of the reference panel) to the diagonal elements of the estimated correlation matrix. To avoid the detrimental effects of SNPs of low imputation accuracy, for the joint testing we retain only eQTL SNPs having the imputation information above a user-selectable threshold (0.3 by default).

### 2.3 Testing for the joint effect of eQTL/functional SNPs

To test for the joint effect of eQTL/functional SNPs known to affect the expression of a gene, JEPEG was designed to rely solely on the (univariate) measured and imputed summary statistics. Based on the database-derived functional category information, JEPEG first groups eQTL/functional SNPs affecting the same gene into the aforementioned six categories: (i) PFS, (ii) TFBS, (iii) miRNA Structure, (iv) miRNA Target, (v) uncategorized cis-eQTLs and (vi) uncategorized trans-eQTLs. These functional SNPs can belong to one or more categories/genes simultaneously. A simple method for estimating the joint effect of all eQTLs associated with a gene might be to combine all eQTL association statistics regardless of their functional category. However, such an approach may result in a saturated statistical model with a large number of degrees of freedom (df), i.e. the number of all eQTLs associated with a gene. When the pairwise LD is elevated, it leads to statistical power loss (Bacanu *et al*., 2008; Chapman *et al*., 2003). To avoid a large number of df for the resulting test statistic (while simultaneously assessing the contribution of each functional category to the overall signal), we pool together statistics of all SNPs from the same functional category in a single synthetic category score. This score is a weighted sum of the *Z*-scores associated with the SNPs in the functional category. The weighted sums of all functional categories influencing a gene are subsequently combined in a gene-level statistic by using a Mahalanobis-type statistic, which takes into account their multivariate correlation (as estimated from a relevant reference panel).

In more detail, let *Z* be the vector of *Z*-scores for *m* SNPs functionally associated with the gene under investigation, *Y* be the diagonal matrix of the square root of imputation information for the *m* functional SNPs, *S* be the weight matrix, as derived from the SNP annotation database, for the *m* functional SNPs belonging to the *k* functional categories. *S* consists of *m* column vectors representing weight scores of the *k* functional categories per SNP, which are precalculated on the basis of the consensus of results from diverse prediction methods (Section 1 in Supplementary Data) and stored in the JEPEG annotation database. To downweight SNPs with low imputation information, based on *Y* and *S*, we compute the adjusted weight matrix by accounting for the imputation information of the SNPs: *W = SY*. Let *∑_G_* be the correlation matrix of SNP genotypes, e.g. as estimated from a reference panel, *U* be the vector of weighted sum of *Z*-scores by category (i.e. the synthetic scores) and *∑_U_* be the variance–covariance/correlation matrix of *U*. Then, in mathematical notation:$U\;=\;WZ\text{and}\sum_{U}=\;W\;\sum_{Z}W^{\text{T}},$where *∑_Z_* is the covariance/correlation matrix of *Z*. Given that, under the null hypothesis of no association between genotype and trait (*H*_0_), *Z* is asymptotically distributed as a multivariate normal with a zero mean vector and covariance matrix *∑_G_*, it follows that:$\sum_{U}=\;W\;\sum_{G}W^{\text{T}}.$Due to LD, *∑_G_* might be close to singular, which results in unstable estimation of the gene-based test statistic. Thus, to stabilize JEPEG statistic, we add the DIST ridge adjustment to the diagonal elements of *∑_G_*. Based on the synthetic scores of all functional categories affecting the gene and their correlation structure, JEPEG computes an omnibus gene-level test as$T\;=\;U^{T}\sum_{U}{}^{-\text{1}}U,$which, under *H*_0_, is asymptotically distributed as a central *χ^2^* statistic with *k* df. The two-tailed *P*-values associated with the normalized *U* can be used as a *post* *hoc* measure to evaluate the contribution of each functional category to the omnibus gene signal.

#### Adjustment for the background enrichment of GWAS signals

Large GWAS/meta-analyses [such as Psychiatric Genomics Consortium (PGC)] harbor abundant small or moderate association signals not reaching significance thresholds across the entire genome. Thus, even when a gene is not related to the trait, due to the background enrichment of the entire genome, we have elevated chances to detect a signal in such a gene. Intuitively, background enrichment makes the sum of squares of the univariate statistics to behave like a non-central *χ^2^* variable. Consequently, for large studies, it is more desirable (and conservative) to assess the statistical significance/*P*-value of *T* after adjusting for background enrichment. In more detail, let *N* be the total number of tested genes and *T_i_* and *k_i_* be the JEPEG test statistic and df of the *ith* gene, respectively. The enrichment adjusted *P*-value of the *ith* gene is then obtained under the assumption that *T_i_* follows a non-central *χ^2^* distribution with *k_i_* df and a non-centrality parameter per df $\lambda =\max(\sum_{i=1}^{N}\left(\frac{T_{i}-k_{i}}{k_{i}}\right)/N,\;0)$ (Bacanu *et al*., 2014).

### 2.4 Reference population

In the current version, we have the capability of using as reference populations 1KG Europeans (*n* = 379), Asians (*n* = 286), Africans (*n* = 246) and Americans (*n* = 181). These panels were obtained from 1KG Phase I release version 3 database, by including only biallelic SNPs, indels and structural variants with two or more allelles. These reference panels are available for both imputation (DIST) and gene-level testing (JEPEG) modules. Future iterations of the software will be able to (i) use larger reference panels and (ii) be applied to cosmopolitan cohorts.

### 2.5 Assessment of Type I error rate of JEPEG

To estimate the Type I error rates of JEPEG, we simulated (under *H*_0_) 100 realistic Illumina 1 M (http://www.illumina.com) GWAS summary datasets for both continuous and binary phenotypes. For each simulation, the genotypic data were obtained by randomly drawing with replacement 10 000 subjects from UK10K dataset (http://www.uk10k.org) and retaining as GWAS (measured) SNPs only those found on Illumina 1 M chip. The continuous phenotype was simulated as random standard normal variables, and the binary phenotype was obtained by randomly assigning case status to 5000 subjects and control status to the remaining subjects. The summary statistics were obtained by testing for association between SNP and phenotype using linear/logistic regression. We applied JEPEG to the 100 simulated *H*_0_ summary dataset from each phenotype type and estimated the empirical Type I error rates. To evaluate the robustness of the proposed method when the LD matrix of the study cohort and reference population is not perfectly matched, we used the more ethnically diverse 1KG Europeans as a reference population to analyse the UK10K-derived data.

### 2.6 Assessment of JEPEG performance

To evaluate the performance of the proposed method, we compared it to the commonly used univariate GWAS methods and, at default settings, with VErsatile Gene-based Association Study (VEGAS), a broadly used gene-based test for association (http://gump.qimr.edu.au/VEGAS/) (Liu *et al*., 2010). VEGAS uses as a test statistic the sum of univariate χ^2^ of SNPs within a gene and assesses its statistical significance using an empirical *H*_0_ distribution simulated from a multivariate normal distribution with LD matrix of the SNPs as a covariance matrix. We applied the earlier methods to four real summary datasets: (i) PGC1 bipolar disorder (BD) (Sklar *et al*., 2011), (ii) schizophrenia (SCZ) (Ripke *et al*., 2011), (iii) major depressive disorder (MDD) (Sullivan *et al*., 2013) cohorts and (iv) anorexia nervosa cohort from Genetic Consortium For Anorexia Nervosa (GCAN) (Boraska *et al*., 2014). Before the applied analyses, we converted all four summary datasets to National Center for Biotechnology Information (NCBI) build 37 (hg19) using liftOver (Hinrichs *et al*., 2006). 1KG Europeans data was used as the reference panel for JEPEG.

To limit the increase in Type I error rates of JEPEG due to certain genes being non-causal but very close to GWAS peaks, we adjusted all JEPEG gene level statistics for background enrichment. Enrichment-adjusted JEPEG gene-level *P* values were subsequently adjusted for multiple testing by using the false discovery rate (FDR) procedure. Genes with FDR-adjusted JEPEG *P*-value (*q*-value) < 0.05 were deemed significant. We also deemed as suggestive genes having non-significant *q*-values below 0.16, i.e. the *P*-value threshold corresponding to Akaike Information Criterion. Due to the difficulty of assigning df to their statistics, VEGAS gene statistics were not adjusted for background enrichment but they were adjusted for multiple testing using FDR.

## 3 Results

**Fig. 2.**
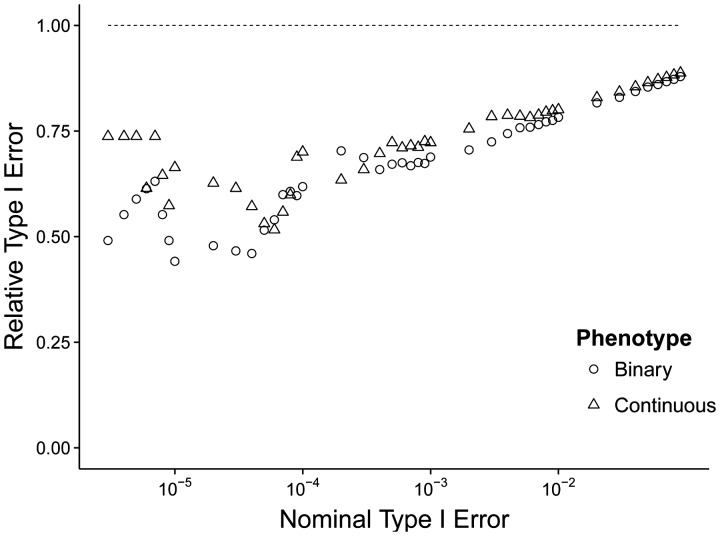
JEPEG relative Type I error rate (the empirical Type I error rate divided by the nominal Type I error rate) as a function of the nominal Type I error rate, (log 10 scale) and the phenotype used. The dashed line denotes the nominal threshold for the relative Type I error rate

While *H*_0_ summary datasets were simulated based on the fairly homogenous samples from UK10K and analysed using the multi-ethnic 1KG Europeans reference panel, JEPEG still controls the Type I error rates at or below the nominal level (Fig. 2). The results suggest that JEPEG with the ridge correction is reasonably robust to (non-African) intracontinental LD variations.

In PGC1 BD cohort, out of 13 552 genes with reliable functional information, we detected 10 significant and 4 suggestive signals (Table 1). The most significant gene (*q*-value = 3 × 10^−4^) was *RASGEF1A* (10q11.21), which was never reported to be associated with BD. For this gene, JEPEG database contained functional annotation information for only six trans-eQTL SNPs on chromosome 6, with the most significant residing near *SYNE1,* a gene already detected univariately in PGC1 BD meta-analysis (Sklar *et al*., 2011). The second most significant gene was the *SYNE1* gene (*q*-value = 8 × 10^−4^) itself, for which the statistic was obtained based on 38 nearby eQTL SNPs. Five of the significant genes belong to *ITIH* cluster on chromosome 3, which also encompasses two other suggestive genes. It is notable that *ITIH* cluster did not yield any significant SNP signal in PGC1 BD (or SCZ) but was detected univariately only in the (much larger) combined analysis of PGC1 BD and SCZ (Sklar *et al*., 2011). For the same cohort, VEGAS detected 27 significant and 8 suggestive gene signals out of 17 789 genes (Supplementary Table S1). Significant signals were obtained from six gene regions, where, except the marginally significant chr2 *LMAN2L* and chr19 *NFIX* (albeit VEGAS standard errors are orders of magnitude larger than the small estimated *P*-values)*,* also detected by JEPEG. VEGAS did not detect *RASGEF1A*. We note that while most regions were detected by both multivariate methods, JEPEG appears to fine-map these regions by returning smaller gene lists.

**Table 1.** JEPEG results for PGC1 BD

Gene	Chr	Start	End	*χ^2^*	df	*P*	*q*	*n*	Top Categ (*P*)	Top SNP (*P*)
Significant Genes (*q* < 0.05)										
* RASGEF1A*	10	43,194,533	43,266,919	31.2	1	2.30 × 10^−8^	0.0003	6	TRN (2.30 × 10^−8)^	rs9371601 (4.33 × 10^−9^)
* SYNE1*	6	152,121,684	152,637,399	35.0	3	1.19 × 10^−7^	0.0008	38	PFS (1.06 × 10^−7^)	rs214976 (2.47 × 10^−8^)
* DDN*	12	48,995,150	48,999,305	26.5	2	1.76 × 10^−6^	0.0064	2	CIS (8.85 × 10^−7^)	rs10783299 (2.53 × 10^−7^)
* ****GLT8D1***	3	52,694,484	52,706,083	22.7	1	1.90 × 10^−6^	0.0064	21	CIS (1.90 × 10^−6^)	rs2251219 (5.45 × 10^−7^)
* ****GNL3***	3	52,685,920	52,694,497	22.0	1	2.69 × 10^−6^	0.0066	1	PFS (2.69 × 10^−6^)	rs2289247 (8.55 × 10^−7^)
* ****SNORD19***	3	52,689,240	52,689,315	21.9	1	2.92 × 10^−6^	0.0066	1	PFS (2.92 × 10^−6^)	rs11177 (9.35 × 10^−7^)
* ****ITIH1***	3	52,777,586	52,792,068	23.7	2	7.12 × 10^−6^	0.0138	4	PFS (4.05 × 10^−6^)	rs1042779 (1.90 × 10^−6^)
*C15orf53*	15	38,696,598	38,700,038	19.7	1	8.98 × 10^−6^	0.0152	1	PFS (8.98 × 10^−6^)	rs7165988 (3.21 × 10^−6^)
* PC*	11	66,848,522	66,958,376	18.0	1	2.20 × 10^−5^	0.0332	1	TFB (2.20 × 10^−5^)	rs3741194 (8.57 × 10^−6^)
* ****MUSTN1***	3	52,833,115	52,835,219	17.2	1	3.32 × 10^−5^	0.0450	2	PFS (3.32 × 10^−5^)	rs4687657 (1.02 × 10^−5^)
Suggestive Genes (0.05 < *q* < 0.16)										
* NEK4*	3	52,710,780	52,770,949	16.1	1	6.10 × 10^−5^	0.0751	2	PFS (6.10 × 10^−5^)	rs1029871 (8.97 × 10^−7^)
* ANKRD18DP*	3	198,057,531	198,080,671	15.6	1	8.01 × 10^−5^	0.0904	6	TRN (8.01 × 10^−5^)	rs1077352 (4.80 × 10^−5^)
* PCSK7*	11	117,199,836	117,232,525	18.6	2	9.29 × 10^−5^	0.0968	4	TFB (2.23 × 10^−4^)	rs201598301 (1.08 × 10^−4^)
* TUBA1B*	12	49,127,782	49,131,521	14.9	1	1.14 × 10^−4^	0.1105	1	TFB (1.14 × 10^−4^)	rs1057725 (5.20 × 10^−5^)

**Bold** denotes significant genes not reported in PGC1 but in the supersets of PGC1. Underline denotes newly found non MHC significant genes, with solid underline for genes with more than one eQTL SNP and 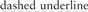 for genes with only one non-significant eQTL. Gene, HUGO gene name; Chr, chromosome number; Start, start position of gene; End, end position of gene; *χ^2^*, JEPEG test statistic; df, degrees of freedom; *P*, *P*-value before background enrichment adjustment; *q*, background enrichment adjusted FDR *q*-value; *n*, number of eQTLs associated with gene; Top Categ (*P*), top functional category and its *P*-value; Top SNP (*P*), SNP ID of top eQTL and its *P*-value; PFS, Protein Function/Structure; TFB, TFBS; STR, miRNA Structure; TAR, miRNA Target; CIS, cis-eQTLs; TRN, trans-eQTLs.

For the SCZ cohort, out of 13 420 genes with functional information, 18 and 13 genes harbored significant and suggestive signals, respectively (Table 2). However, only six significant genes were outside the major histocompatibility complex (MHC) region (chr6: 28–33.5 Mb), which has been associated with SCZ in many Caucasian GWAS (Purcell *et al*., 2009; Stefansson *et al*., 2009). We underscore the detection of a significant signal for *MAD1L1* (*q*-value = 0.01). This gene was not identified in univariately in PGC1 SCZ, but was detected in a larger PGC1 follow-up study, which included additional Swedish cohorts (Ripke *et al*., 2013). We also detected 3 significant and 3 suggestive gene signals from *ITIH* cluster. We also note the strong significant SCZ signals in *NKAPL* and *ZKSCAN4*, which were the only MHC genes harboring significant signals in a Han Chinese SCZ GWAS (Yue *et al*., 2011). Meanwhile, for the same data, VEGAS detected only 3 significant (*ABCC12*, *SRCAP* and *ZNF629*) and 2 suggestive (*PHKG2* and *ZNF681*) gene signals out of 17 704 genes (Supplementary Table S2). We mention that none of the genes with significant VEGAS signal are located within LD independent SCZ association regions from the latest PGC SCZ Stage 2 study (Ripke *et al*., 2014).

**Table 2.** JEPEG results for PGC1 SCZ. (see Table 1 for background and notation.)

Gene	Chr	Start	End	$\chi^{2}$	df	*P*	*q*	*n*	Top Categ (*P*)	Top SNP (*P*)
Significant Genes (*q* < 0.05)										
*BTN3A2*	6	26,365,159	26,378,320	39.4	3	1.45 × 10^−8^	0.0007	56	TRN (1.67 × 10^−9^)	rs17693963 (1.56 × 10^−10^)
*HLA-DRB5*	6	32,517,374	32,530,229	36.5	3	5.90 × 10^−8^	0.0010	58	TRN (8.07 × 10^−9^)	rs116115875 (9.90 × 10^−7^)
*NKAPL*	6	28,259,297	28,260,958	32.6	2	8.26 × 10^−8^	0.0010	3	TRN (4.40 × 10^−8^)	rs1679709 (9.39 × 10^−9^)
*BTN2A1*	6	26,457,904	26,476,621	30.0	2	3.00 × 10^−7^	0.0027	7	PFS (6.64 × 10^−8^)	rs13195401 (3.41 × 10^−7^)
*HLA-A*	6	29,942,470	29,945,884	27.0	2	1.34 × 10^−6^	0.0073	15	CIS (2.05 × 10^−7^)	rs114197794 (8.69 × 10^−9^)
*HIST1H2BL*	6	27,807,479	27,807,931	23.1	1	1.51 × 10^−6^	0.0073	1	PFS (1.51 × 10^−6^)	rs200484 (4.56 × 10^−7^)
*HIST1H2BPS1*	6	25,731,728	25,732,166	22.5	1	2.10 × 10^−6^	0.0086	1	TAR (2.10 × 10^−6^)	rs9461209 (6.51 × 10^−7^)
***MAD1L1***	7	1,815,792	2,232,948	25.6	2	2.82 × 10^−6^	0.0099	9	PFS (3.30 × 10^−6^)	rs1801368 (1.07 × 10^−6^)
*OR12D3*	6	29,373,423	29,375,291	25.5	2	2.83 × 10^−6^	0.0099	30	CIS (9.03 × 10^−7^)	rs114071887 (2.59 × 10^−7^)
*ZKSCAN4*	6	28,244,626	28,259,252	21.1	1	4.32 × 10^−6^	0.0119	2	PFS (4.32 × 10^−6^)	rs9986596 (3.94 × 10^−9^)
***MUSTN1***	3	52,833,115	52,835,219	20.7	1	5.37 × 10^−6^	0.0133	2	PFS (5.37 × 10^−6^)	rs4687657 (3.65 × 10^−6^)
*OR2B2*	6	27,911,185	27,912,396	19.7	1	8.98 × 10^−6^	0.0193	4	PFS (8.98 × 10^−6^)	rs34788973 (6.31 × 10^−9^)
***ITIH4***	3	52,812,990	52,830,701	19.6	1	9.50 × 10^−6^	0.0193	1	TRN (9.50 × 10^−6^)	rs2276817 (3.41 × 10^−6^)
*ZNF323*	6	28,324,737	28,337,366	24.5	3	1.98 × 10^−5^	0.0375	16	CIS (1.12 × 10^−6^)	rs2859365 (2.45 × 10^−6^)
*VKORC1L1*	7	65,873,270	65,959,563	18.1	1	2.08 × 10^−5^	0.0375	1	CIS (2.08 × 10^−5^)	rs4962347 (8.04 × 10^−6^)
*HIST1H2AL*	6	27,865,329	27,865,798	17.9	1	2.34 × 10^−5^	0.0375	3	TFB (2.34 × 10^−5^)	rs200981 (1.70 × 10^−7^)
***GLT8D1***	3	52,694,484	52,706,083	17.6	1	2.78 × 10^−5^	0.0408	21	CIS (2.78 × 10^−5^)	rs3733047 (1.06 × 10^−5^)
*TCP10L*	21	32,574,841	32,585,535	17.3	1	3.12 × 10^−5^	0.0429	1	PFS (3.12 × 10^−5^)	rs9622 (1.25 × 10^−5^)
Suggestive genes (0.05 < *q* < 0.16)										
*BTN3A1*	6	26,402,237	26,415,216	20.3	2	3.99 × 10^−5^	0.0565	5	PFS (1.26 × 10^−4^)	rs41266839 (1.78 × 10^−7^)
*PTGES*	9	129,738,336	129,753,065	16.6	1	4.66 × 10^−5^	0.0565	1	CIS (4.66 × 10^−5^)	rs6592945 (1.95 × 10^−5^)
*LIN28B*	6	104,950,467	105,083,332	16.5	1	4.95 × 10^−5^	0.0570	2	TRN (4.95 × 10^−5^)	rs17195211 (3.26 × 10^−4^)
*MIR8064*	3	52,846,463	52,846,552	16.0	1	6.26 × 10^−5^	0.0680	1	STR (6.26 × 10^−5^)	rs4687672 (2.69 × 10^−5^)
*ZSCAN31*	6	28,324,737	28,337,366	15.3	1	9.01 × 10^−5^	0.0916	3	PFS (9.01 × 10^−5^)	rs853678 (1.06 × 10^−9^)
*KATNAL2*	18	46,917,602	47,102,243	14.8	1	1.20 × 10^−4^	0.1150	1	PFS (1.20 × 10^−4^)	rs7233515 (5.48 × 10^−5^)
*ITIH1*	3	52,777,586	52,792,068	17.8	2	1.40 × 10^−4^	0.1292	4	PFS (6.68 × 10^−5^)	rs678 (4.08 × 10^−5^)
*PTK7*	6	43,076,268	43,161,720	14.5	1	1.42 × 10^−4^	0.1248	4	PFS (1.42 × 10^−4^)	rs34764696 (6.37 × 10^−5^)
*SNORD19*	3	52,689,240	52,689,315	14.5	1	1.42 × 10^−4^	0.1248	1	PFS (1.42 × 10^−4^)	rs11177 (6.61 × 10^−5^)
*CUL9*	6	43,182,175	43,224,587	17.3	2	1.77 × 10^−4^	0.1501	6	TFB (4.40 × 10^−5^)	rs2273709 (5.98 × 10^−6^)
*ZBED4*	22	49,853,849	49,890,078	17.2	2	1.84 × 10^−4^	0.1501	2	PFS (9.01 × 10^−4^)	rs910799 (4.99 × 10^−4^)
*SCARNA3*	1	175,968,397	175,968,540	13.9	1	1.91 × 10^−4^	0.1501	10	TRN (1.91 × 10^−4^)	rs12220941 (8.81 × 10^−5^)
*ZKSCAN8*	6	28,141,910	28,159,472	13.7	1	2.10 × 10^−4^	0.1511	1	TFB (2.10 × 10^−4^)	rs17774663 (1.01 × 10^−4^)

For PGC1 MDD and GCAN studies, neither multivariate method yields any significant findings. While disappointing, our findings closely mirror the univariate results. An increase in sample size for the two disorders might help increase signal detection power for all methods.

On a computation node with 4x Intel Xeon 6 core 2.67-GHz processor and 64 GB of RAM, the single core JEPEG analyses for any of the four summary datasets required slightly under 2 h of running time and less than 8 GB of peak memory usage. The web-based VEGAS software (http://gump.qimr.edu.au/VEGAS/) at default settings required around 1 day of computation time for each summary dataset.

## 4 Conclusions

In this article, we propose JEPEG, a new software/method for testing the joint effects on trait for SNPs functionally associated with a gene. The proposed method (i) imputes unmeasured functional SNPs, (ii) pools in a synthetic variable the information of all (measured and imputed) SNPs in the same functional category, (iii) to obtain an omnibus gene statistic, combines these synthetic variables in a Mahalanobis-type test and (iv) provides single functional category statistics, which can be used to identify the categories driving the overall omnibus signal. We use realistic simulated datasets, to show that JEPEG controls the Type I error rates at or below nominal rates. The application of the method to PGC1 BD and SCZ datasets suggests that JEPEG has the potential to improve both gene detection and fine mapping of challenging regions, such as MHC for SCZ and *ITIH* cluster for BD (SCZ).

The argument that the joint eQTL testing might substantially increase detection power is strongly supported by three key findings from the applied analyses. First, we identified at least one novel candidate gene for BD, *RASGEF1A*, based on its trans-eQTL SNPs. Due to trans-eQTLs being generally considered less reliable, the evidence for this gene should be viewed with caution. However, we note that *RASGEF1A* is already known to be implicated in a peripheral neural disorders (Hirschprung’s disease) (Fernandez *et al*., 2012). Second, we detected both significant BD and SCZ signals in *ITIH* cluster, which was univariately uncovered only in a much larger *combined* analysis of PGC1 BD and SCZ cohorts (Sklar *et al*., 2011). Third, in PGC1 we detected a significant SCZ signal for *MAD1L1,* which was not identified by VEGAS and was univariately detected only in a larger superset of PGC1 (Ripke *et al*., 2013).

The practical applications suggest that JEPEG has the potential to aid fine-mapping of challenging regions. For instance, some of the largest MHC signals for SCZ were in *NKAPL* and *ZKSCAN4*, which were the only MHC genes with significant signals in a Han Chinese SCZ cohort (Yue *et al*., 2011). Even more, while VEGAS detected 12 BD signals in *ITIH* cluster, the strength of JEPEG BD signals suggests that the five JEPEG genes in *ITIH* cluster are more likely to be functionally involved in BD etiology. The three JEPEG SCZ signals in the *ITIH* cluster support the hypothesis of a pleiotropic effect on both disorders. If we are further willing to assume that the same *ITIH* genes might predispose to both disorders, the intersection of SCZ and BD signals might be used to further narrow the list of candidate genes to just *GLT8D1* and *MUSTN1*.

Given its novel multivariate testing of functional SNPs, JEPEG is a complementary tool to the commonly used univariate GWAS approach and agnostic multivariate approaches like VEGAS. Our approach will augment the performance of these methods for certain biologically plausible causal models that are less suitable to univariate/agnostic detection, e.g. genes with multiple functional SNPs jointly acting on a trait. Due to basing its inference solely on summary statistics, the proposed method can be used even when subject-level genotype data is not available. Even more, because the LD structure used by JEPEG is unaffected by the relatedness between samples, it can be used in its current form to analyse summary data coming from large family studies.

We plan to further develop and update JEPEG along three main directions. First, we plan to upgrade the SNP annotation database by extending the number of SNPs and their functional annotations. For instance, we plan to add to the database variants specific to (i) 1KG non-Caucasian cohorts, (ii) UK10K (http://www.uk10k.org) and (iii) X-linked eQTL SNPs. We will also continuously update the functional categorization and SNP weights based on the latest available detection tools and practical evidence from larger empirical studies. Second, based on the available scientific evidence, we will add functional SNPs from other potentially relevant tissues/cell types (e.g. lymphocytes and monocytes). Third, we plan to add additional functional categories. The current version relies heavily on functional categories for which we are able to predict the direction and magnitude of the effect of SNP's reference allele on gene expression. This feature was convenient because it allowed for pooling of Z-scores within each functional category in a one df statistic. However, while expedient, this is not a requirement for our method. When the direction of the allelic effect on gene expression is hard to predict, we can still employ the computationally more complex weighted sum of *χ^2^* statistics within such categories (Davies, 1980). Thus, by using such a weighted *χ^2^* statistics approach, we plan to extend JEPEG to include other important functional categories such as methylation tagging SNPs, Dnase hypersensitivity sites and histone marks.

We note that JEPEG and its summary statistics-based imputation module (DIST) offer best performance when the pairwise LD matrix of the study cohort and reference panel is identical. Thus, when the study and reference population are not ethnically well matched or the study cohort is multiethnic, JEPEG might provide suboptimal results including some spurious signals. However, our extensive simulation experiments (e.g. Fig. 2) suggest that JEPEG might be reasonably robust to (non-African) intracontinental LD patterns of variation.

JEPEG is written in C++ with open-source numerical libraries. JEPEG software along with database of eQTL SNPs, reference panels, usage instructions and examples are publicly available at http://code.google.com/p/jepeg. For more details on (or usage of) the direct imputation method employed internally by JEPEG, please see http://code.google.com/p/dist. We welcome user critiques and suggestions for improvement regarding the method itself and the functional SNP database.

## Funding

This work was supported by R25DA026119 (D.L.), MH100560 (B.P.R. and S.A.B.), 1P50AA022537 (S.A.B. and B.P.R.) and AA022717 (V.S.W., V.I.V. and S.A.B.).

*Conflict of interest*: none declared.

## Supplementary Material

Supplementary Data
