# Supplementary material for: JEPEG: a summary statistics based tool for gene-level joint testing of functional variants
Source: Bioinformatics. 2014 Dec 12;31(8):1176–82. doi: 10.1093/bioinformatics/btu816 (PMC4393522; doi:10.1093/bioinformatics/btu816)
Supplement: Supplementary Data [file supp_31_8_1176__index.html]

JEPEG: a summary statistics based tool for gene-level joint testing of functional variants — JEPEG: a summary statistics based tool for gene-level joint testing of functional variants — JEPEG: a summary statistics based tool for gene-level joint testing of functional variants — Supplementary Data 

# JEPEG: a summary statistics based tool for gene-level joint testing of functional variants

## Supplementary Data

files

**Files in this Data Supplement:**

- Supplementary Data - docx file
