# Supplementary material for: JEPEG: a summary statistics based tool for gene-level joint testing of functional variants
Source: Bioinformatics. 2014 Dec 12;31(8):1176–82. doi: 10.1093/bioinformatics/btu816 (PMC4393522; doi:10.1093/bioinformatics/btu816)
Supplement: Supplementary Data [file supp_btu816_jepeg_supplementary_data.docx]

**Supplementary Data**

**1. Development of SNP annotation database**

For mapping and in silico-based analysis of probable function, we start with a list of 38,209,239 SNPs extracted from Phase 1 of 1000 genome data (1KG). In the first iteration of the database we include only 34,138,445 SNPs polymorphic in Caucasians. We retained initially only functional SNPs predicted to affect gene expression in brain. The brain-expressed genes were selected using publicly available projects such as: Database of Genotypes and Phenotypes (Mailman et al., 2007) and the Genotype-Tissue expression (Lonsdale et al., 2013). Batch Coordinate Conversion (liftover tool, UCSC Genome Browser) was used to ensure that base location of each SNP is consistent across different annotation builds (HG19/GRCh37) (Hinrichs et al., 2006). The eQTL database is based on polymorphic variants in the 379 Europeans from 1KG phase 1 release, which is estimated to capture ≈ 98% of the human genetic variants. Many of these variants are predicted to cause frame shifts, premature stop codons or disturb splice site patterns, all with detrimental effect on gene function. To ensure that only high-quality data enter our database we 1) include variants with Phred quality scores ≥ 30 and 2) assess consistency of the variants’ calls between 1KG and UK10K for individuals represented in both databases. Effect sizes of the biological categories selected for use in the initial database design are estimated from the Encyclopedia of DNA Elements (ENCODE) project. ENCODE currently lists genetic, expression and methylation data on a wide variety of cell types and tissues (ENCODE Project Consortium, 2004; ENCODE Project Consortium et al., 2012). We predict variants' effect sizes from directly observable instances in the ENCODE data obtained for cortical neurons as well as in whole brain, prefrontal cortex and hypothalamus. Bioinformatic predictions on the effect sizes are based on a consensus approach using the prediction scores identified by two or more programs.

**Protein Function/Structure (PFS)** - a SNP can affect the protein function and structure by altering the amino acid residues through its position within a codon. While these may not be strictly considered an eQTL (e.g. they may affect protein function without affecting mRNA expression), these functional variants were included in the database because they dramatically affect the product of genes. Each SNP is assessed using multiple in silico prediction methods, e.g. Gerp++, Sift, LJB, and Polyphen (Adzhubei et al., 2010; Davydov et al., 2010; Kumar et al., 2009; Liu et al., 2011) and its effect on protein structure is predicted through: 1) levels of evolutionary constraint, 2) effect on the codon functionality and 3) effect on amino acid hydrophobicity (Bateman et al., 2000). Using summaries of each program, a likelihood score, ranging from 0 (neutral) to 1 (highly deleterious), was generated for each SNP. An example of highly deleterious SNPs is polymorphism generating a premature stop codon; the likelihood score for such SNP is expected to be a maximum of one. For this functional category, JEPEG uses the likelihood score to weight SNP summary statistics.

**Transcription Factor Binding Sites (TFBS)-** Another mechanism by which a SNP affects gene expression is through the disruption of a transcription factor binding site (TFBS). Transcription factors (TF) affect gene expression through binding to specific sites within the promoter region of a gene (Stormo, 2000; Wingender et al., 1997). To assess which SNPs had a differential effect on TF binding, a two-step algorithm was devised. First, a position–specific scoring matrix (PSSM) was used on multiple sequence alignments (MSA) of human/mouse/rat genomes to identify conserved genomic regions. Conserved sites were screened based of the respective Z-scores of each matrix against known TF databases and only those sites meeting a score threshold +/- 1.96 (e.g. highly conserved sites) were retained. Second, an individual SNP’s allelic effect was determined by the magnitude of change in the Z-score between the PSSMs of the TFBS variants containing each of the two SNP alleles (Reddy et al., 2012; Spivakov et al., 2012). For TFBS, JEPEG uses the Z-scores to weight SNP summary statistics.

**Micro RNA (miRNA) Structure -** MiRNAs are small (~ 22 bases) non-coding RNAs affecting gene function mainly negatively (Ambros, 2004; Ambros et al., 2003; Ambros and Lee, 2004). There has been increasing evidence linking the disruption of miRNA biogenesis by SNPs (Clop et al., 2006; Georges et al., 2006; Mishra et al., 2008; Ripke et al., 2011). SNPs disruptions have been noted in the precursor and mature miRNA sequences as well as in the proteins participating in processing of these sequences (Sun et al., 2009; Zhou et al., 2012). At the time of database creation, SNPs were mapped against all known human (MiRbase v.19) miRNA precursor and mature sequences (N = 1600; 2042, respectively) and miRNAs found to contain a SNP in the hairpin or the mature sequences were further analyzed. To determine SNP allele effect on the precursor secondary structure’s free energy, miRNA precursor sequences containing each alternate SNP allele were assessed using RNAfold (McCaskill, 1990; Zuker and Stiegler, 1981). SNPs were declared to have a differential effect on the miRNA secondary structure if they introduced a change of _Δ_G ≥ 1ºC. For this functional category, JEPEG uses _Δ_G to weight SNP summary statistics.

**miRNA-mRNA Interaction -** In addition to determining SNP effect on miRNA secondary structure, SNPs affecting miRNA-mRNA interaction are also included. It has been shown that the “seed” region (~6-8 bases long) at the 5’ end of the miRNA molecule is important for down-regulation of gene target expression. Thus, SNPs affecting the seed region can either eliminate a target site or create a new one (Georges et al., 2006; Glinsky, 2008; Hiard et al., 2010; Landi et al., 2008; Saunders et al., 2007; Sethupathy and Collins, 2008). To assess SNP allelic effect on miRNA gene target interaction, here, first all targets for each known miRNAs were predicted using a consensus approach of four commonly used programs (MiRanda, Pita, TargetScanS, and Pictar) (John et al., 2004; Kertesz et al., 2007; Lewis et al., 2005; Lewis et al., 2003). It has been suggested that a consensus approach for miRNA target prediction across multiple algorithms reduces the high number of false positive findings occurring in in-silico predicted miRNA/mRNA interactions (Wang and Wang, 2006; Xia et al., 2009; Yu et al., 2007; Zhang and Verbeek, 2010). A Mahalanobis distance score (D^2^) across each of the four programs was used to filter high quality targets (Xu et al., 2012). Allelic effect of SNPs mapping onto these targets was evaluated using RNAfold to calculate binding energy between the miRNA and the proposed target gene site. A difference in free energy of one degree or higher was taken to indicate a differential effect. Similar to the other miRNA functional category, JEPEG uses _Δ_G to weight SNP summary statistics.

**Uncategorized eQTL** - Under the classic definition, a SNP polymorphism whose genotype is correlated with expression of a mRNA transcript will be considered an eQTL. Two types of eQTLs, i.e. cis and trans, have been identified based on the relative location to a mRNA transcript or gene of interest (Montgomery et al., 2010; Pickrell et al., 2010). Cis-eQTLs map close to the gene of origin (<1 megabase) and trans-eQTLs are found further away from the gene, sometimes on different chromosomes (Veyrieras et al., 2008). In our database we included in the cis-eQTL category, only the cis-eQTLs which were not categorized in any of the above functional categories. Trans-eQTLs have been noted to be more tissue dependent than cis-eQTLs (de et al., 2012; Gerrits et al., 2009). Multiple experimental methods have been used to identify eQTLs including RNA-Seq and microarray. The goal of eQTL analysis is to identify active genome regions which then can be used to provide a link between the genetic (association) and molecular (expression) data. This can further be used to strengthen the genetic data and to provide a causal direction for observed expression differences. eQTL studies also help to identify a set of genes more directly involved in a disease phenotype, from those whose expression is a result of unrelated biological processes. SNPs from the 1KG were mapped to a publically available database, which is formulated from a meta-analysis of seven different eQTL experiments on a range of tissues and cell types including whole brain, lymphocytes and monocytes (Myers et al., 2007; Xia et al., 2012). However, for the current version of SNP annotation database, we use only functional SNPs relevant to whole brain. The eQTLs in this database were first identified using the R package (Matrix EQTL) and based on an additive linear regression model, considering multiple covariates such as gender and age. The scores reported for each mapped SNP from the database represent the False Discovery Rate (FDR) calculated separately for cis and trans eQTLs. For uncategorized eQTL category, JEPEG uses -log10(FDR) to weight SNP summary statistics. Given that trans-eQTLs are considered to be less reproducible compared to cis-eQTLs, the future version JEPEG will internally penalize the weight score for trans-eQTLs, i.e. -log10(FDR), by dividing them by a factor of 2.

| **Table S1.** VEGAS results for PGC1 BD. Gene, HUGO gene name; Chr, chromosome number; Start, start position of gene; End, end position of gene; Test, VEGAS test statistic; *n*, number of SNPs; *p*, p-value; *q*, FDR q-value; Top SNP, top SNP ID; Top SNP *p*, top SNP p-value. | | | | | | | | | |
| --- | --- | --- | --- | --- | --- | --- | --- | --- | --- |
| **Gene** | **Chr** | **Start** | **End** | **Test** | ***n*** | ***p*** | ***q*** | **Top SNP** | **Top SNP *p*** |
| **Significant Genes (*q* < 0.05)** | | | | | | | | | |
| DHH | 12 | 47769472 | 47774869 | 568.8239 | 31 | 5.00E-07 | 0.002965 | rs7296288 | 8.39E-08 |
| MLL2 | 12 | 47699024 | 47735374 | 611.5599 | 35 | 5.00E-07 | 0.002965 | rs7296288 | 8.39E-08 |
| RHEBL1 | 12 | 47744734 | 47750042 | 600.9833 | 31 | 5.00E-07 | 0.002965 | rs7296288 | 8.39E-08 |
| LMBR1L | 12 | 47777189 | 47790947 | 577.2801 | 32 | 1.00E-06 | 0.003234 | rs7296288 | 8.39E-08 |
| PRKAG1 | 12 | 47682321 | 47698859 | 336.1654 | 29 | 1.00E-06 | 0.003234 | rs10875914 | 5.63E-07 |
| GNL3 | 3 | 52694975 | 52703550 | 638.609 | 38 | 2.00E-06 | 0.003234 | rs11130315 | 3.72E-06 |
| ITIH1 | 3 | 52786647 | 52801117 | 1126.807 | 82 | 2.00E-06 | 0.003234 | rs736408 | 1.22E-06 |
| ITIH3 | 3 | 52803823 | 52818065 | 1138.971 | 87 | 2.00E-06 | 0.003234 | rs736408 | 1.22E-06 |
| NEK4 | 3 | 52719840 | 52779991 | 1304.737 | 84 | 2.00E-06 | 0.003234 | rs736408 | 1.22E-06 |
| DDN | 12 | 47675199 | 47679355 | 307.2606 | 28 | 2.00E-06 | 0.003234 | rs10875914 | 5.63E-07 |
| TUBA1B | 12 | 47807832 | 47811571 | 523.6865 | 30 | 2.00E-06 | 0.003234 | rs7296288 | 8.39E-08 |
| PBRM1 | 3 | 52554407 | 52694906 | 1469.322 | 102 | 3.00E-06 | 0.004447 | rs2251219 | 2.93E-06 |
| MUSTN1 | 3 | 52842176 | 52844260 | 1054.34 | 96 | 5.00E-06 | 0.006842 | rs736408 | 1.22E-06 |
| GLT8D1 | 3 | 52703543 | 52715088 | 635.0248 | 38 | 6.00E-06 | 0.007116 | rs11130315 | 3.72E-06 |
| SPCS1 | 3 | 52714896 | 52717237 | 593.2125 | 35 | 6.00E-06 | 0.007116 | rs11130315 | 3.72E-06 |
| ITIH4 | 3 | 52822045 | 52839734 | 1143.755 | 99 | 7.00E-06 | 0.007783 | rs736408 | 1.22E-06 |
| SYNE1 | 6 | 152484514 | 153000227 | 3391.927 | 776 | 1.10E-05 | 0.011511 | rs9371601 | 4.27E-08 |
| C11orf80 | 11 | 66268782 | 66367563 | 385.7445 | 31 | 1.20E-05 | 0.011859 | rs10896135 | 8.46E-06 |
| LRFN4 | 11 | 66381451 | 66384522 | 272.9684 | 24 | 1.70E-05 | 0.015916 | rs7930203 | 1.17E-05 |
| LOC440957 | 3 | 52545660 | 52549626 | 554.9923 | 50 | 2.00E-05 | 0.016942 | rs2251219 | 2.93E-06 |
| NT5DC2 | 3 | 52533442 | 52544110 | 541.8683 | 59 | 2.00E-05 | 0.016942 | rs2251219 | 2.93E-06 |
| TMEM110 | 3 | 52848937 | 52906587 | 1025.688 | 109 | 2.30E-05 | 0.01853 | rs736408 | 1.22E-06 |
| RCE1 | 11 | 66367458 | 66370579 | 271.1046 | 19 | 2.40E-05 | 0.01853 | rs7930203 | 1.17E-05 |
| PC | 11 | 66372572 | 66482423 | 443.1786 | 50 | 2.50E-05 | 0.01853 | rs7930203 | 1.17E-05 |
| CACNB3 | 12 | 47498778 | 47508991 | 331.7644 | 57 | 2.90E-05 | 0.020635 | rs11168751 | 1.80E-05 |
| NFIX | 19 | 12967583 | 13070610 | 249.9173 | 49 | 3.40E-05 | 0.02306 | rs11085829 | 4.03E-06 |
| LMAN2L | 2 | 96735393 | 96769528 | 242.0376 | 32 | 3.50E-05 | 0.02306 | rs6746896 | 2.33E-06 |
| **Suggestive Genes (0.05 < *q* < 0.16)** | | | | | | | | | |
| TUBA1A | 12 | 47864849 | 47869128 | 238.757 | 24 | 0.000105 | 0.066709 | rs1991427 | 7.22E-05 |
| NUDT1 | 7 | 2248382 | 2257306 | 358.4815 | 59 | 0.000119 | 0.072996 | rs12699820 | 4.18E-05 |
| BCL11B | 14 | 98705377 | 98807575 | 758.4458 | 201 | 0.000127 | 0.075307 | rs941519 | 0.000168 |
| SNX8 | 7 | 2261164 | 2320625 | 367.1781 | 65 | 0.000137 | 0.078616 | rs13245097 | 3.81E-05 |
| ATXN1 | 6 | 16407321 | 16869700 | 1807.229 | 648 | 0.000161 | 0.089501 | rs2299061 | 9.51E-05 |
| ADCY6 | 12 | 47446241 | 47469087 | 272.1225 | 48 | 0.000172 | 0.092718 | rs11168751 | 1.80E-05 |
| FTSJ2 | 7 | 2240451 | 2248359 | 358.9225 | 66 | 0.000193 | 0.100979 | rs2398668 | 4.26E-05 |
| WNT1 | 12 | 47658502 | 47662746 | 224.1852 | 37 | 0.000253 | 0.128589 | rs10875914 | 5.63E-07 |

| **Table S2.** VEGAS results for PGC1 SCZ. See Table S1 for background. | | | | | | | | | |
| --- | --- | --- | --- | --- | --- | --- | --- | --- | --- |
| **Gene** | **Chr** | **Start** | **End** | **Test** | ***n*** | ***p*** | ***q*** | **Top SNP** | **Top SNP *p*** |
| **Significant Genes (*q* < 0.05)** | | | | | | | | | |
| ABCC12 | 16 | 46674384 | 46738182 | 91.07092 | 31 | 5.00E-07 | 0.004426 | rs16945872 | 0.002446 |
| SRCAP | 16 | 30617962 | 30658951 | 30.00539 | 9 | 5.00E-07 | 0.004426 | rs4889505 | 0.03682 |
| ZNF629 | 16 | 30697270 | 30706024 | 39.99421 | 10 | 1.00E-06 | 0.005901 | rs6565208 | 0.003603 |
| **Suggestive Genes (0.05 < *q* < 0.16)** | | | | | | | | | |
| PHKG2 | 16 | 30667237 | 30676183 | 22.80418 | 7 | 2.80E-05 | 0.109765 | rs4889505 | 0.03682 |
| ZNF681 | 19 | 23713836 | 23733533 | 60.23894 | 30 | 3.10E-05 | 0.109765 | rs7507855 | 0.06937 |

Reference List

Adzhubei,I.A. et al. (2010) A method and server for predicting damaging missense mutations. *Nat. Methods*, 7, 248-249.

Ambros,V. (2004) The functions of animal microRNAs. *Nature*, 431, 350-355.

Ambros,V. et al. (2003) A uniform system for microRNA annotation. *RNA*, 9, 277-279.

Ambros,V. and Lee,R.C. (2004) Identification of microRNAs and other tiny noncoding RNAs by cDNA cloning. *Methods Mol. Biol.*, 265, 131-158.

Bateman,A. et al. (2000) The Pfam Protein Families Database. *Nucleic Acids Res.*, 28, 263-266.

Clop,A. et al. (2006) A mutation creating a potential illegitimate microRNA target site in the myostatin gene affects muscularity in sheep. *Nat. Genet.*, 38, 813-818.

Davydov,E.V. et al. (2010) Identifying a high fraction of the human genome to be under selective constraint using GERP++. *PLoS Comput. Biol.*, 6, e1001025.

de,J.S. et al. (2012) Expression QTL analysis of top loci from GWAS meta-analysis highlights additional schizophrenia candidate genes. *European journal of human genetics : EJHG*, 20, 1004-1008.

ENCODE Project Consortium. (2004) The ENCODE (ENCyclopedia Of DNA Elements) Project. *Science*, 306, 636-640.

ENCODE Project Consortium et al. (2012) An integrated encyclopedia of DNA elements in the human genome. *Nature*, 489, 57-74.

Georges,M. et al. (2006) Polymorphic microRNA-target interactions: a novel source of phenotypic variation. *Cold Spring Harb. Symp. Quant. Biol.*, 71, 343-350.

Gerrits,A. et al. (2009) Expression quantitative trait loci are highly sensitive to cellular differentiation state. *PLoS genetics*, 5, e1000692.

Glinsky,G.V. (2008) An SNP-guided microRNA map of fifteen common human disorders identifies a consensus disease phenocode aiming at principal components of the nuclear import pathway. *Cell. Cycle*, 7, 2570-2583.

Hiard,S. et al. (2010) Patrocles: a database of polymorphic miRNA-mediated gene regulation in vertebrates. *Nucleic Acids Res.*, 38, D640-D651.

Hinrichs,A.S. et al. (2006) The UCSC Genome Browser Database: update 2006. *Nucleic Acids Research*, 34, D590-D598.

John,B. et al. (2004) Human MicroRNA targets. *PLoS Biol*, 2, e363.

Kertesz,M. et al. (2007) The role of site accessibility in microRNA target recognition. *Nat Genet*, 39, 1278-1284.

Kumar,P., Henikoff,S. and Ng,P.C. (2009) Predicting the effects of coding non-synonymous variants on protein function using the SIFT algorithm. *Nat. Protoc.*, 4, 1073-1081.

Landi,D. et al. (2008) Polymorphisms within micro-RNA-binding sites and risk of sporadic colorectal cancer. 29, 579-584.

Lewis,B.P., Burge,C.B. and Bartel,D.P. (2005) Conserved seed pairing, often flanked by adenosines, indicates that thousands of human genes are microRNA targets. *Cell*, 120, 15-20.

Lewis,B.P. et al. (2003) Prediction of mammalian microRNA targets. *Cell*, 115, 787-798.

Liu,X., Jian,X. and Boerwinkle,E. (2011) dbNSFP: a lightweight database of human nonsynonymous SNPs and their functional predictions. *Hum. Mutat.*, 32, 894-899.

Lonsdale,J. et al. (2013) The Genotype-Tissue Expression (GTEx) project. *Nat Genet*, 45, 580-585.

Mailman,M.D. et al. (2007) The NCBI dbGaP database of genotypes and phenotypes. *Nat. Genet.*, 39, 1181-1186.

McCaskill,J.S. (1990) The equilibrium partition function and base pair binding probabilities for RNA secondary structure. *Biopolymers*, 29, 1105-1119.

Mishra,P.J., Banerjee,D. and Bertino,J.R. (2008) MiRSNPs or MiR-polymorphisms, new players in microRNA mediated regulation of the cell - Introducing microRNA pharmacogenomics. 7, 853-858.

Montgomery,S.B. et al. (2010) Transcriptome genetics using second generation sequencing in a Caucasian population. *Nature*, 464, 773-777.

Myers,A.J. et al. (2007) A survey of genetic human cortical gene expression. *Nature genetics*, 39, 1494-1499.

Pickrell,J.K. et al. (2010) Understanding mechanisms underlying human gene expression variation with RNA sequencing. *Nature*, 464, 768-772.

Reddy,T.E. et al. (2012) Effects of sequence variation on differential allelic transcription factor occupancy and gene expression. *Genome Res.*, 22, 860-869.

Ripke,S. et al. (2011) Genome-wide association study identifies five new schizophrenia loci. *Nat. Genet.*, 43, 969-976.

Saunders,M.A., Liang,H. and Li,W.H. (2007) Human polymorphism at microRNAs and microRNA target sites. *Proc. Natl. Acad. Sci. U. S. A.*, 104, 3300-3305.

Sethupathy,P. and Collins,F.S. (2008) MicroRNA target site polymorphisms and human disease. 24, 489-497.

Spivakov,M. et al. (2012) Analysis of variation at transcription factor binding sites in Drosophila and humans. *Genome Biol.*, 13, R49.

Stormo,G.D. (2000) DNA binding sites: representation and discovery. *Bioinformatics*, 16, 16-23.

Sun,G. et al. (2009) SNPs in human miRNA genes affect biogenesis and function. *RNA*, 15, 1640-1651.

Veyrieras,J.B. et al. (2008) High-resolution mapping of expression-QTLs yields insight into human gene regulation. *PLoS genetics*, 4, e1000214.

Wang,X.W. and Wang,X.H. (2006) Systematic identification of microRNA functions by combining target prediction and expression profiling. 34, 1646-1652.

Wingender,E. et al. (1997) TRANSFAC, TRRD and COMPEL: towards a federated database system on transcriptional regulation. *Nucleic Acids Res.*, 25, 265-268.

Xia,K. et al. (2012) seeQTL: a searchable database for human eQTLs. *Bioinformatics (Oxford, England)*, 28, 451-452.

Xia,W., Cao,G. and Shao,N. (2009) Progress in miRNA target prediction and identification. *Sci. China C. Life. Sci.*, 52, 1123-1130.

Xu,W. et al. (2012) Coding SNPs as intrinsic markers for sample tracking in large-scale transcriptome studies. *BioTechniques*, 52, 386-388.

Yu,Z.B. et al. (2007) Aberrant allele frequencies of the SNPs located in microRNA target sites are potentially associated with human cancers. 35, 4535-4541.

Zhang,Y. and Verbeek,F.J. (2010) Comparison and integration of target prediction algorithms for microRNA studies. *J. Integr. Bioinform.*, 7, 10-2010.

Zhou,Y. et al. (2012) Evaluation of Six SNPs of MicroRNA Machinery Genes and Risk of Schizophrenia. *J. Mol. Neurosci., 49, 594-599*

Zuker,M. and Stiegler,P. (1981) Optimal computer folding of large RNA sequences using thermodynamics and auxiliary information. *Nucleic Acids Res.*, 9, 133-148.
